# The Long Isoform of Intersectin-1 Has a Role in Learning and Memory

**DOI:** 10.3389/fnbeh.2020.00024

**Published:** 2020-02-25

**Authors:** Nakisa Malakooti, Melanie A. Pritchard, Feng Chen, Yong Yu, Charlotte Sgambelloni, Paul A. Adlard, David I. Finkelstein

**Affiliations:** ^1^Florey Institute of Neuroscience and Mental Health, University of Melbourne, Parkville, VIC, Australia; ^2^Department of Biochemistry and Molecular Biology, Faculty of Medicine, Nursing & Health Sciences, Monash University, Clayton, VIC, Australia

**Keywords:** intersectin-1, down syndrome, learning, memory, cognition, dendritic spines, cell signaling

## Abstract

Down syndrome is caused by partial or total trisomy of chromosome 21 and is characterized by intellectual disability and other disorders. Although it is difficult to determine which of the genes over-expressed on the supernumerary chromosome contribute to a specific abnormality, one approach is to study each gene in isolation. This can be accomplished either by using an over-expression model to study increased gene dosage or a gene-deficiency model to study the biological function of the gene. Here, we extend our examination of the function of the chromosome 21 gene, *ITSN1*. We used mice in which the long isoform of intersectin-1 was knocked out (ITSN1-LKO) to understand how a lack of the long isoform of *ITSN1* affects brain function. We examined cognitive and locomotor behavior as well as long term potentiation (LTP) and the mitogen-activated protein kinase (MAPK) and 3′-kinase-C2β-AKT (AKT) cell signaling pathways. We also examined the density of dendritic spines on hippocampal pyramidal neurons. We observed that ITSN1-LKO mice had deficits in learning and long term spatial memory. They also exhibited impaired LTP, and no changes in the levels of the phosphorylated extracellular signal-regulated kinase (ERK) 1/2. The amount of phosphorylated AKT was reduced in the ITSN1-LKO hippocampus and there was a decrease in the number of apical dendritic spines in hippocampal neurons. Our data suggest that the long isoform of ITSN1 plays a part in normal learning and memory.

## Introduction

The most common intellectual disability, Down syndrome (trisomy 21), has an incidence of 1 in 700 live births (Irving et al., 2008). Intersectin-1 (*ITSN1)* is one of the 219 protein-coding genes on chromosome 21 (HUGO Gene Nomenclature Committee, 2017) and is over-expressed in Down syndrome (Pucharcós et al., 1999), suggesting that ITSN1 may contribute to the phenotypes associated with this disorder. ITSN1 has two major protein isoforms as a result of differential splicing: a long 200 KDa form (ITSN1-L); and a short 140 KDa form (ITSN1-S). The long isoform is predominantly expressed in neuronal cells but is also expressed in the eye, heart, white blood cells and kidney tissue while the short isoform is expressed in all tissues (Hussain et al., 1999; Ma et al., 2003; Yu et al., 2008). The long isoform has three additional protein domains at the C-terminus which are involved in calcium-binding, the regulation of cell signaling, actin cytoskeleton rearrangement (Tsyba et al., 2011), the function of ion channels (Khanna et al., 2007) and rapid neurotransmission (Sakaba et al., 2013).

ITSN1 acts as a scaffold, with different proteins binding to its domains to affect their functions (reviewed in Hunter et al., 2013). ITSN1 has been shown by us and others to be involved in endocytosis/exocytosis (Yu et al., 2008; Pechstein et al., 2010; Tsyba et al., 2011). Indeed, through its role in endocytosis of clathrin-coated vesicles, ITSN1 was shown to be involved in the fusion and formation processes essential for the control of nerve cell communication at pre- and post-synapses (Sakaba et al., 2013) and have a primary function in sustained rapid neurotransmission *via* the replenishment of release-ready synaptic vesicles (Sakaba et al., 2013). ITSN1 also has a regulatory role in a growing number of signal transduction pathways (Wang and Shen, 2011; Wong et al., 2012; Hunter et al., 2013), which among others, include the mitogen-activated protein kinase (MAPK) cell signaling pathway (Adams et al., 2000; Predescu et al., 2007) and neuron survival through the 3′-kinase-C2β-AKT pathway (Das et al., 2007).

A role in dendritic spine morphogenesis has been attributed to ITSN1. The long isoform, ITSN1-L, is known to associate *via* its guanine nucleotide exchange factor (GEF) activity with neural Wiskott-Aldrich syndrome protein (N-WASP), which constitutively activates the Rho-family GTPase Cdc42, leading to actin polymerization and spine morphogenesis (Hussain et al., 2001; Irie and Yamaguchi, 2002; Pechstein et al., 2010). Further, a study by Thomas et al. (2009) showed that intersectin-1 co-localizes with F-actin at dendritic spines and that there was an increased number of filopodia, together with a decreased number of mushroom spines in intersectin knocked-down neurons, suggesting that intersectin has an effect on the morphogenesis of dendritic spines.

Thus, ITSN1 seems to connect a number of cellular functions at the nerve terminal, including synaptic vesicle recycling and the regulation of actin dynamics and cell signaling. Therefore, higher-order activities of the brain such as learning and memory may also depend on ITSN1. To investigate such a role for ITSN1 and to advance our understanding of the biological function of the long isoform and its possible contribution to the intellectual deficits associated with DS, we embarked upon the current study. Here, we tested locomotor activity, cognition, cell signaling pathways, synaptic activity (long term potentiation, LTP) and hippocampal dendritic spine density in both wild type (WT) and ITSN1-LKO mice. We report that ITSN1-LKO mice exhibit long-term memory deficits, impaired LTP and AKT signaling in the hippocampus as well as a reduction in the number of apical dendritic spines on hippocampal pyramidal neurons. Taken together, our results suggest that ITSN1-L may have a role in hippocampal-dependent functions such as learning and memory.

## Materials and Methods

### Mice

ITSN1-LKO mice were generated as described by Yu et al. (2008). A targeting vector was constructed to delete exon 32 of the ITSN1 gene, creating the frameshift required to generate ITSN1-LKO embryonic stem (ES) cell clones. Briefly, fragments containing exon 32 using ITSN1-specific DNA probes were screened for in a lambda mouse genomic library from strain 129SV/J. Flanking exon 32 with *loxP* sites allowed the deletion of the protein *via* a new in-frame stop codon in the adjacent downstream codon. A neomycin resistance cassette was located between the *loxP* sites and a Herpes simplex virus-1 thymidine kinase gene was positioned at the end of 3’-ends of the targeting vector to allow the selection in ES cells. Correctly targeted ES cell clones were selected and injected into 129SV/J mice blastocysts. Resultant chimeric mice were mated with 129SV/J to produce heterozygous ITSN1-LKO mice. Heterozygous mice were mated and sibling pairs used to establish homozygous and wild-type control lines for further use.

All the mice were genotyped by Transgenix using PCR oligonucleotide primers:

- LK2429f (5′-CTgACgTTgggAAATACgATgAgA-3′)
- LK3743f (5′-gATAgACTCTAACCTgTAAgCCAg-3′)
- LK2918r (5′-gATgTCgCCAATCATCTTCACCg-3′)

The PCR programs used were: an initial denaturation step at 94 oC for 5 min, followed by 40 cycles each of denaturation (94°C for 30 s), annealing (61°C for 30 s) and extension (68°C for 30 s), and the final extension step for 7 min.

ITSN1-LKO mice were characterized by Yu (2010). The female ITSN1-LKO had a lower body weight and smaller body size at the time of weaning compared with the WT, but not the male mice. There were no differences between the gross brain structures including whole brain volume, hippocampus volume, cortical thickness and the ratio of hippocampus volume to brain volume. All ITSN1-LKO were viable, fertile and apparently healthy.

### Ethics Statement

All animal experiments were approved by the Florey Institute of Neuroscience and Mental Health Animal Ethics Committee and were conducted in accordance with the Australian Code of Practice for the Care and Use of Animals for Scientific Purposes as described by the National Health and Medical Research Council of Australia. ITSN1-LKO mice were generated as described (Yu et al., 2008). Young mice (9–12 weeks) and older mice (6 months old) were used for experiments. All ITSN1-LKO and WT mice were genotyped by Transnetyx Inc. (Cordova, TN, USA). Animals were housed in a 12 h light/dark cycle and had free access to food and water.

### Behavioral Studies

#### Morris Water Maze (MWM)

Cognition was tested using the Morris water maze (MWM), a test designed to examine hippocampal function. Experiments were performed on a cohort of 11–12 male mice per genotype at 9–12 weeks and 6 months of age. Prior to the MWM test (day 0), a swim test was conducted where the mice were allowed to swim for 90 s. The distance traveled (cm) by each mouse and the velocity (cm/s) was measured to ensure that there were no differences between the groups that might account for any apparent differences in the performance of the task. Over 6 days of experiments spatial and long term memory were tested. In a circular pool (1.5-m diameter) filled with warm water (23–24°C), a 10 cm escape platform (submerged 1.5 cm below the surface) was placed in a fixed position (NW quadrant, 25 cm away from the pool wall). The water was made opaque by the addition of non-toxic paint and visual cues were mounted on the room walls at fixed positions. Animals were subjected to four training sessions per day (90 s/trial) and were placed randomly in the four different starting positions: NE; NW; SE; or SW quadrants. Using the visual cues around the room, mice were able to find the submerged platform and escape the water. Each attempt lasted for 90 s. An attempt was considered successful if the mouse remained on the platform for 5 s. At the end of 90 s, unsuccessful mice were placed on the platform for 5 s as a reminder of the location of the platform. The time taken by each mouse to find the platform in each trial was recorded over 6 days. On day 7, a probe trial test was conducted. The hidden platform was removed and the mice were allowed to swim for 90 s. The time that each mouse spent in the NW quadrant was recorded where the escape platform had previously been located. A cue trial test was also conducted to test the visual capacity and level of motivation of each mouse to find a visible platform. Two consecutive 60-s trials started in two separate quadrants were conducted. The movements of the mice were recorded and analyzed with Ethovision 3.0 tracking system software (Wageningen, The Netherlands).

#### Y-Maze

A Y-maze, adopted from the original radial arm maze (Olton, 1987), using a two-trial recognition memory test, was conducted to assess short term (working) memory. Experiments were performed on a cohort of 11–12 male mice per genotype at 9–12 weeks and 6 months of age. A maze with three arms of length 29.5 cm, width 7.5 cm, and height 15.5 cm, was used. In the first trial, one arm was blocked and the mice were allowed to explore two arms for 10 min. After 1 h, the partition was removed and the mice were returned to the maze and allowed to explore all the arms for 5 min. The time spent in each arm was recorded using Topscan Lite Tracking software (CleverSys, Reston, VA, USA).

#### Open Field

Experiments were conducted on a cohort of six mice per genotype at 6 months of age. We investigated the locomotor activity of the mice in an open field apparatus. Mice were placed in a rectangular (39 cm long × 29 cm wide × 12 cm high) clear plexiglass box that contained a sensing grid. Horizontal and vertical movements were recorded for 30 min to examine total movement and different qualities of these movements, including the number of rearing which is suggestive of the level of anxiety in response to this novel environment. All the data were collated and analyzed by computer-assisted photo-beam activity system (Truscan 2.0 software, Coulbourne Instruments, Whitehall, PA, USA) to assess locomotor activity and anxiety.

#### Electrophysiological Studies

Experiments were performed on a cohort of five mice per genotype at 9–12 weeks and 6 months of age (2–3 slices per mouse, *n* = 5 mice). A Microelectrode Array (MEA; Multi-Channel System, GmbH, Reutlingen, Germany) was used to measure the long-term potentiation (LTP) in the form of enhanced long-lasting field excitatory postsynaptic potentials (fEPSP) which were induced in the hippocampal CA3–CA1 circuit by the stimulating protocol described below. To measure LTP, horizontal hippocampal slices (300 μm) were prepared with a VT 1200S tissue slicer (Leica, Germany) from 9 to 12 weeks and 6-months-old male mice. Slices were quickly transferred to 34°C artificial CSF (aCSF; composition in mmol/L: 126 NaCl, 2.5 KCl, 2.4 CaCl_2_, 1.3 MgCl_2_, 1.25 NaH_2_PO_4_, 26 NaHCO_3_, and 10 D-glucose) and equilibrated for 90 min. A slice was placed on an MEA chip (60 MEA 200/30 iR-Ti: MCS GnbH, Kevelaer, Germany) and immobilized with a harp slice grid (ALA Scientific Instruments, Farmingdale, NY, USA). The Schaffer-collateral pathway was stimulated with a biphasic current waveform (100 μs) through the selected electrode at 0.033 Hz. Data were collected with LTP-Director and analyzed with LTP-Analyser (Multi-Channel Systems; MCS GmbH, Germany). When stable evoked fEPSPs were detected, the stimulus threshold was determined, and a stimulus strength-evoked response curve (input-output, *I-O curve*) was recorded by gradually increasing stimulus intensity until the maximal EPSP was obtained. Following a 20 min incubation period, slices were continuously stimulated once every 30 s with stimuli that produced 50% of the maximum evoked response for at least 20 min. LTP was induced by tetanic stimulation consisting of 3 trains of 50 pulses at 100 Hz lasting for 500 ms at 20-s intervals. Subsequently, the potentiation of fEPSPs was recorded for 30 min. The peak amplitude of the EPSP was calculated for each evoked response and analyzed.

### Tissue Collection

Mice were killed with an overdose of sodium pentobarbitone (Lethabarb; 100 mg/kg body weight; Virbac., Milperra, NSW, Australia) diluted in 0.9% saline. The mice were perfused *via* the left ventricle with 20 ml ice-cold PBS until blood was flushed away. After perfusion, the brains were removed was micro-dissected to obtain hippocampi, cortices, and cerebellum which were snap-frozen on dry ice.

### Western Blotting

To measure the level of active (phosphorylated) ERK1/2 and AKT we conducted western blotting. Snap-frozen hippocampi were homogenized in ice-cold buffer (10 mM Tris HCL, 150 mM NaCl, 0.1% SDS, 1% sodium deoxycholate, 1%Triton X-100, pH 7.4, supplemented with protease cocktail inhibitor tablets and phosphate inhibitors (Roche, Basel, Switzerland). Protein concentrations of all the samples were quantified using a BCA protein assay kit (Pierce, Thermo Fisher Scientific, Waltham, MA, USA). Protein extract (40 μg) was boiled at 90°C for 5 min in sample buffer (Biorad, USA) and bond-breaker TCEP solution (Thermo Fisher Scientific, Waltham, MA, USA) and resolved on 4–12% Criterion™ XT Bis-Tris Precast Gels (Biorad, Hercules, CA, USA) using Nupage MES SDS running buffer (Invitrogen, Waltham, MA, USA), and transferred by electroblotting onto polyvinylidene fluoride (PVDF) membranes (Immunobilon, Millipore, Burlington, MA, USA) *via* a wet transfer unit (Trans-Blot Electrophoretic Transfer cell, Biorad, USA). Membranes were incubated in blocking buffer (5% (w/v) fat-free milk powder in TBST) for 1 h at room temperature before being incubated with primary antibodies diluted in 0.01% (v/v) Tween 20 in PBS.

Primary antibodies used were mouse anti-p-ERK (1:1,000; ab50011, Abcam, Cambridge, UK), rabbit anti-p-AKT (1:1,000; 13038, Cell Signaling Technology, Danvers, MA, USA) and mouse anti-ERK (1:1,000; ab36991, Abcam, Cambridge, UK) and rabbit anti-AKT (1:1,000; 9272, Cell Signaling Technology, Danvers, MA, USA).

Secondary antibodies were horseradish peroxidase (HRP)-conjugated rabbit anti-mouse (1:2,000 or 1:10,000) or goat anti-rabbit (1:2,000; Dako, Glostrup, Denmark) incubated for 1 h at room temperature. Blots were incubated with Amersham ECL western blotting detection reagent (GE Healthcare, Fairfield, CT, USA) for 1 min to detect HRP activity and the images captured by ImageReader LAS-3,000 and quantified by ImageQuant software (GE Healthcare, Fairfield, CT, USA). Data are presented as the ratios of active (phosphorylated) to total protein for the respective proteins and genotypes.

### Golgi Staining and Quantification of Spine Density

Three 4–6-months-old female mice brains per genotype were used for this assay. To visualize neuronal dendrites, the FD Rapid Golgi kit (MTR Scientific, Germantown, MD, USA) as per the manufacturer’s protocol was used. After processing, brains were frozen on dry ice and were cut using a Leica CM3050 Cryostat (Leica Instruments, Germany) at 100 μm thickness. The sections were dried at RT for 30 min before the staining. The sections were cleared in histosol (HD Scientific, Wetherill Park, NSW, Australia) twice for 5 min each and then placed in xylene (BDH Biomedicals, USA) for 5 min for further clearing before mounting in DPX mounting medium (BDH Biomedicals, USA). Five pyramidal neurons in the hippocampus CA1 region from each mouse were chosen randomly. Spines were counted on five tertiary apical and basal dendrites considering the following criteria: (i) evenness and universal dye impregnation throughout the cell body and the tertiary branches; (ii) the dendrites were not at an angle to the field of view; (iii) the dendrites were well separated from other dendrites. Neurons were visualized on a standard light microscope (Leica Instruments, Germany) with a 100× oil immersion objective and neuronal processes were manually traced onto paper using a drawing tube (MA765, Meiji Techno Company, Japan) attached to the microscope. Dendritic spines were counted and the length of the neuronal processes measured on ImageJ software (NIH) by scanning the manually drawn images onto a computer. Golgi-stained hippocampal brain sections were coded to conceal the identity of the animal’s genotype to avoid bias in assessment (Adlard et al., 2011).

### Statistical Analyses

Data are presented as means ± SEM and were compared by two-tailed student’s *t*-test. MWM escape-latency curves were analyzed using a two-way repeated measure ANOVA followed by a Bonferroni *post hoc* test, with time and genotype as factors. Comparison of average relative escape times and average time spent in the target quadrant at 9–12 weeks and 6 months of age were analyzed using two way ANOVA with a Sidak *post hoc* test. Average LTP was compared using two-way ANOVA with a Sidak *post hoc* test. All statistical analyses were performed using GraphPad Prism 6 software (GraphPad Software, San Diego, CA, USA). The * represents a *p*-value less than 0.05, ** represents a *p*-value less than 0.01 and ***represents a *p*-value less than 0.001.

## Results

### Deletion of the ITSN1 Long Isoform Had No Effect on Locomotor Function

**Figure 1:**
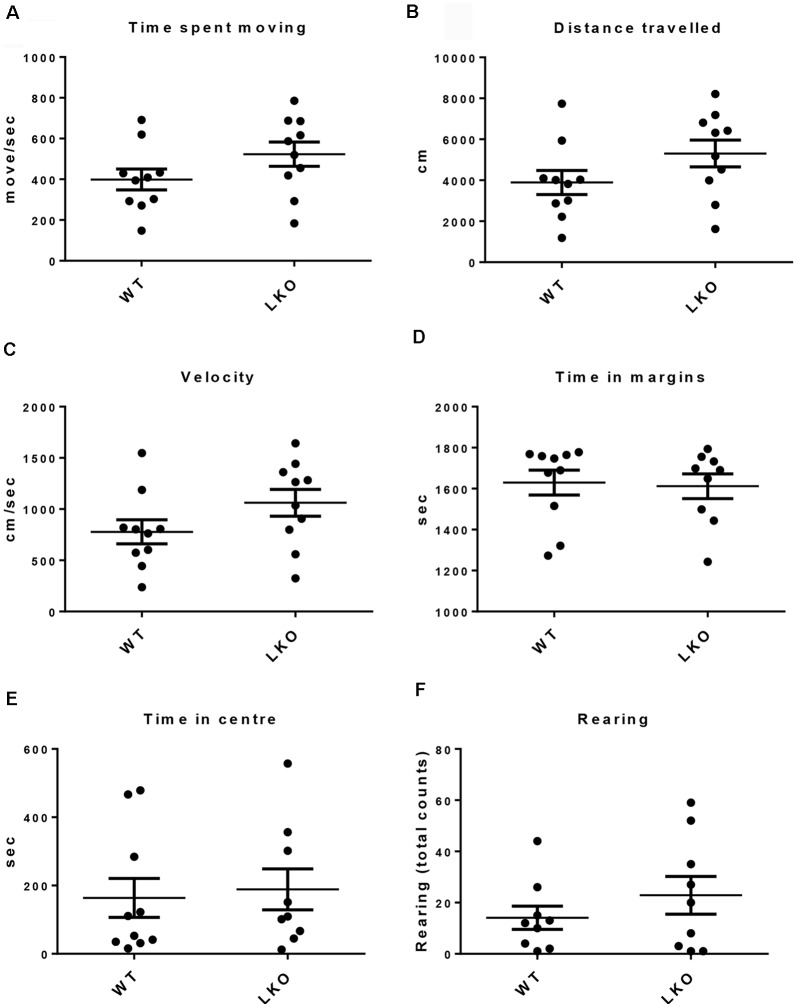
No locomotor function deficits were observed in ITSN1-LKO mice. An open field test showed that there were no differences between ITSN1-LKO and WT mice at 6 months of age. **(A)** Time spent moving (move/s). **(B)** Distance traveled (cm). **(C)** Velocity (cm/s). **(D)** Time spent in the margins (s). **(E)** Time spent in the center (s). **(F)** The number of rears over 30 min. Data are expressed as mean ± SEM. Unpaired two-tailed *t*-test, *n* = 6 per genotype.

To determine if a lack of the ITSN1 long isoform affected locomotor function and to rule out the possibility that differences in locomotor function could influence the interpretation of behavioral tests involving movement, we tested free movement in our cohorts of mice (Beloozerova et al., 2003). We performed an open field test (Stanford, 2007). We observed no differences between the genotypes in several parameters of movement, including time spent moving, distance traveled and velocity (Figures 1A–C). We also measured the time spent in the margins or center of the open field and the number of rearing as indicators of anxiety. We found no significant differences between WT and ITSN1-LKO mice in this regard (Figures 1D–F).

### Deletion of the ITSN1 Long Isoform Impaired Learning and Long-Term Spatial Memory

**Figure 2:**
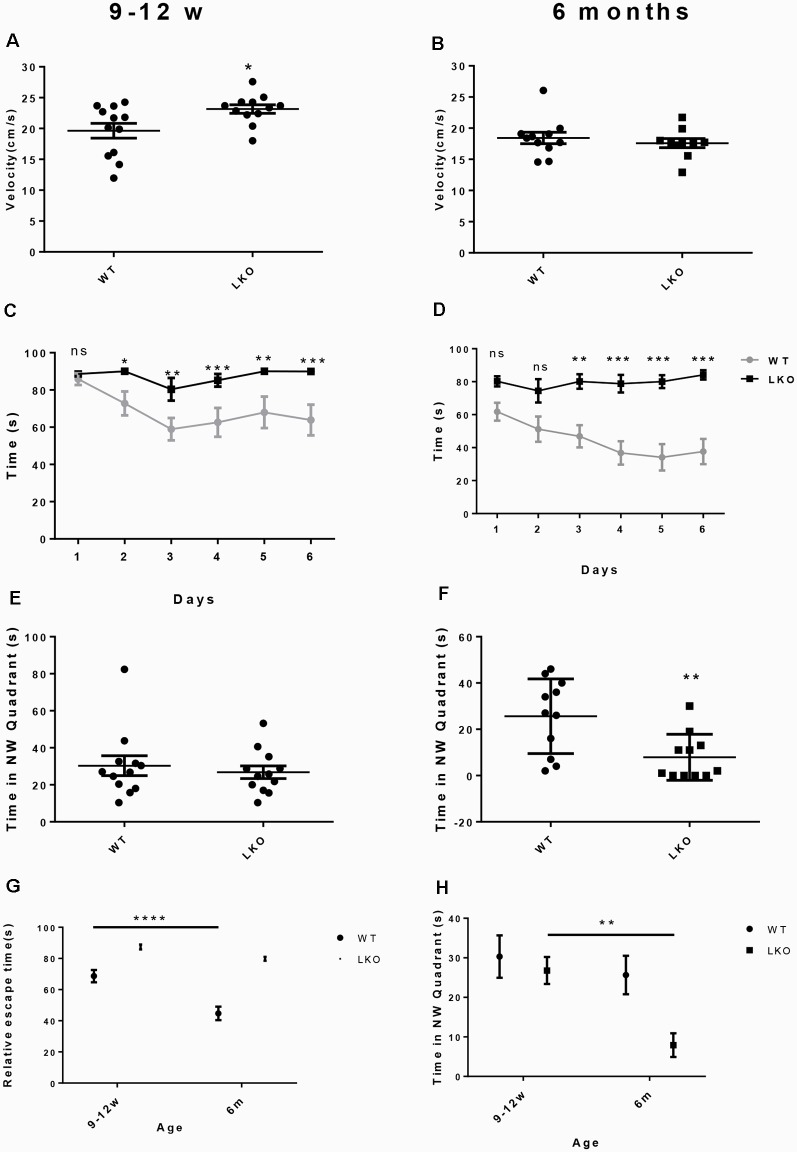
Deletion of the ITSN1 long isoform impaired learning and long term spatial memory in an age-dependent manner. A Morris Water Maze (MWM) was used to assess spatial and long term memory in WT and ITSN1-LKO mice. **(A)** On day 0, there was a significant increase in the swimming velocity of 9–12 weeks old ITSN1-LKO mice compared with age-matched WT controls (*p* = 0.0186, *n* = 12 per group). **(B)** No differences between ITSN1-LKO and WT mice at 6 months of age were observed (*n* = 11 per group, unpaired two-tailed *t*-test). Over 6 days, both young **(C)** and older **(D)** ITSN1-LKO mice took longer to find the hidden platform compared with their WT counterparts, with the difference between WT and KO mice more evident in the older mice. In the probe trial test on day 7, there was no difference in the time spent in the NW quadrant between the genotypes at 9–12 weeks of age **(E)** but at 6 months of age, **(F)** ITSN1-LKO mice did poorly compared with the WT (*p* = 0.0056), implying that loss of long-term memory is age-related in mice deficient for the long isoform of ITSN1. **(G)** Comparison of average escape time as a % of the respective WT at 9–12 weeks and 6 months of age. Two-way ANOVA showed significant main effects of age (*p* < 0.0001) and genotype (*p* < 0.0001) and there was an interaction between age and genotype (*p* = 0.0156). A Sidak’s *post hoc* analysis indicated there was a significant difference between 9–12 weeks and 6-months-old WT mice (*p* < 0.0001). The older WT mice were faster at finding the platform than their younger counterparts, suggesting that learning and spatial memory had improved with age. There was no difference between young and old KO mice. **(H)** Comparison of average time spent in the NW quadrant as a % of the respective WT at 9–12 weeks and 6 months of age. Two-way ANOVA showed significant main effects of age (*p* = 0.0091) and genotype (*p* = 0.0178). A Sidak’s *post hoc* analysis indicated there was a significant difference between 9–12 weeks and 6-months-old LKO mice (*p* = 0.0069). The older KOs spent significantly less time in the quadrant that had previously housed the platform, suggesting that long term memory declined with age in ITSN1-LKO mice. Error bars show means ± SEM, *n* = 11–12 per genotype. *p < 0.05, **p < 0.01, ***p < 0.001, ****p < 0.0001. ns, not significant.

To examine if the long isoform of ITSN1 contributes to learning and long-term spatial memory we conducted a MWM. We first ensured that all mice could swim by measuring swimming velocity. Interestingly, at 9–12 weeks of age, ITSN1-LKO mice swam faster than the WT controls (*p* = 0.0186, *n* = 12 per group, unpaired two-tailed *t*-test; Figure 2A), indicating that any differences observed between the genotypes were not due to swimming ability/motor function. At 6 months of age, there was no difference between the groups (*n* = 11 per group, unpaired two-tailed *t*-test; Figure 2B). Thus, WT and ITSN1-LKO mice swam equally well.

Over the 6 days of testing, we observed significantly higher escape latencies for ITSN1-LKO mice compared with WT mice at both ages: (9–12 weeks old mice, *n* = 12, *p* < 0.05 for day 2, *p* < 0.01 for days 3 and 5, *p* < 0.001 for days 4 and 6); (6-months-old mice, *n* = 12, *p* = 0.0032 for day 1, *p* = 0.0001 for day 2, *p* < 0.0001 for days 4, 5 and 6; Figures 2C,D). These data suggest that both young and older mice have an impaired ability to learn.

On day 7, a probe trial test was conducted to assess the ability of the mice to recall the position of the platform. The hidden platform was removed and the time spent in the target NW quadrant assessed. ITSN1-LKO mice at 9–12 weeks of age spent the same amount of time in the target quadrant as their WT counterparts (Figure 2E). However, 6-months-old ITSN1-LKO mice spent significantly less time in the target quadrant (two-tailed *t*-test, *p* = 0.0056; Figure 2F), suggesting that young ITSN1-LKO had the ability to remember the location of the hidden platform, but older ITSN1-LKO had lost this ability.

Finally, we compared average escape latencies and time spent in the NW target quadrant across the four groups of mice to examine if there were any age-related differences. Interestingly, 6 months-old WT mice were faster at escaping the water compared with their younger counterparts (*p* < 0.0001; Figure 2G) suggesting they were better learners and there appeared to be an age-related decline in memory in the KOs, with 6-months-old KOs performing markedly worse than their 9–12 weeks old counterparts at recalling the whereabouts of the hidden platform (*p* = 0.0069; Figure 2H).

### Loss of the ITSN1 Long Isoform Had No Effect on Short Term Memory

**Figure 3:**
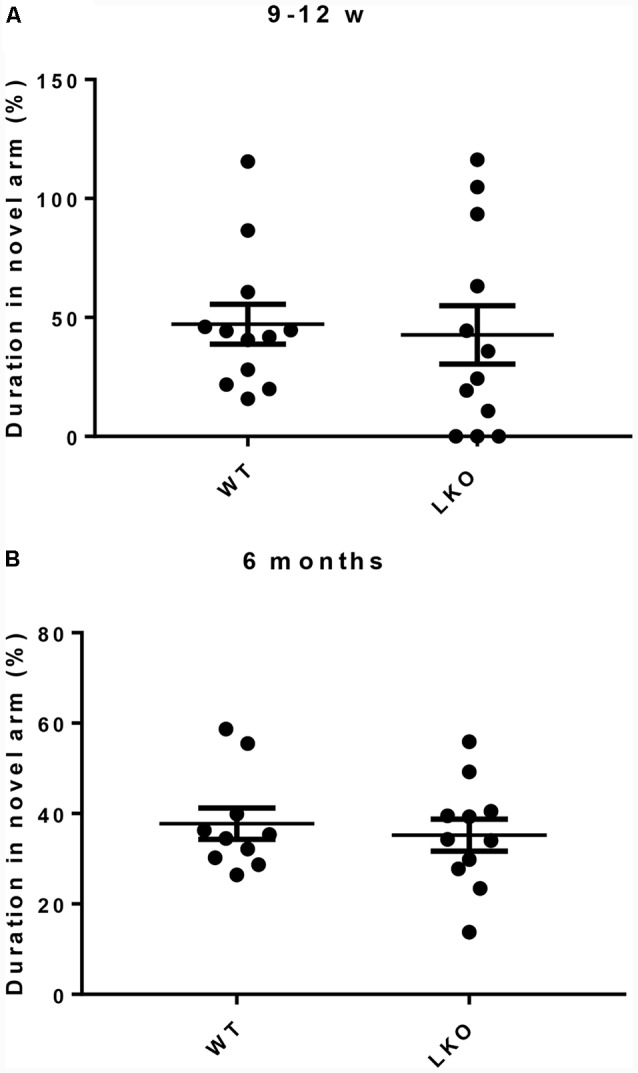
Short term working memory was intact in ITSN1-LKO mice. For both young and old mice, the amount of time spent in the novel arm was equivalent across the genotypes **(A,B)**, suggesting that a lack of the long isoform of ITSN1 had no adverse effect on short term memory. Error bars show means ± SEM, *n* = 11–12 per genotype.

To investigate if ITSN1 contributes to short term (working) memory, a *Y*-maze test was conducted (Adlard et al., 2010). This test uses the natural tendency of mice to explore novel locations. Mice with an intact short term memory tend to explore the novel arm more than the known arms. Young and older mice of both genotypes spent equal time exploring the novel arm (Figures 3A,B). These data suggest that ITSN1-LKO mice do not exhibit short term memory impairment.

### Loss of the ITSN1 Long Isoform Reduced Long-Term Potentiation (LTP)

**Figure 4:**
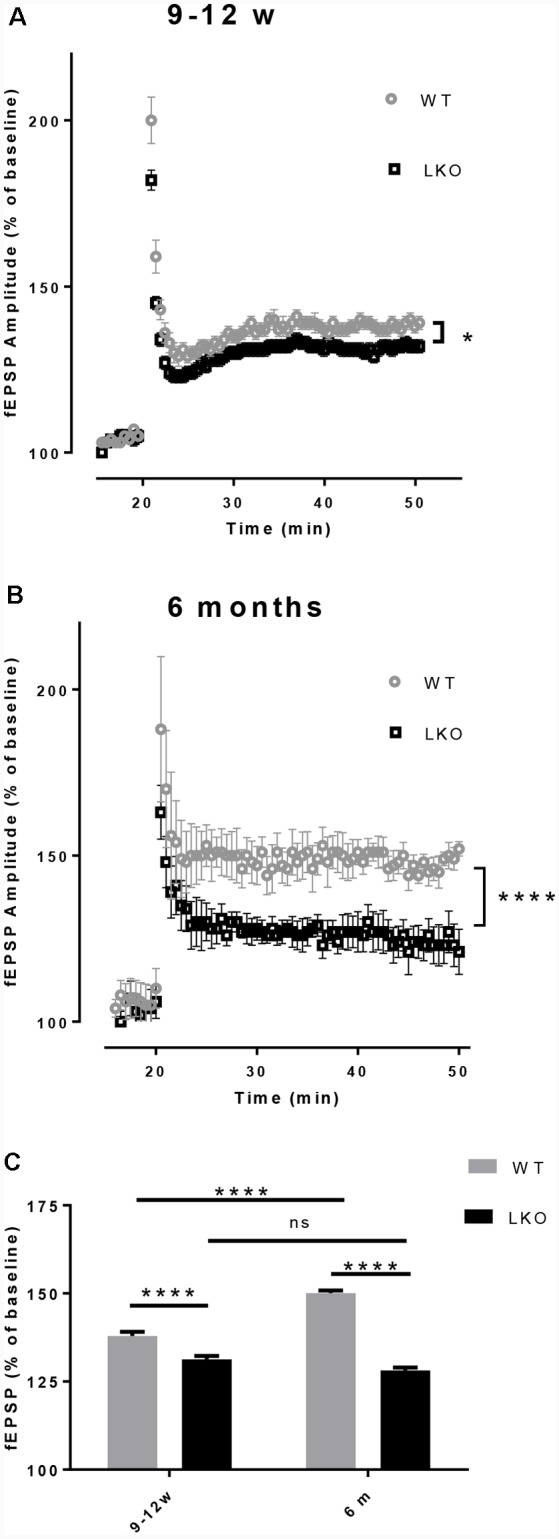
Deletion of ITSN1-L impaired hippocampal long term potentiation (LTP). Field excitatory synaptic potentials (fEPSPs) in the hippocampal CA1 region were recorded to measure LTP and are expressed as mean percentage ± SEM change over baseline. **(A)** LTP was diminished in 9–12 weeks old ITSN1-LKO mice compared with WT controls (*p < 0.05, *n* = 5 per genotype). **(B)** LTP was also reduced in ITSN1-LKO mice at 6 months of age (****p < 0.0001, 2–3 slices per mouse, *n* = 5 per genotype). Although the initial amplitude of the potentiation resulting from tetanic stimulation was similar between genotypes, after three trains of high-frequency stimulation to induce LTP, WT mice sustained this potentiation for a greater period of time than ITSN1-LKO mice at both ages, with a more pronounced decline apparent in knockout mice at 6 months of age. A two-tailed *t*-test for each time point was used for statistical analysis. **(C)** Comparison of the average change in LTP as a % of the respective WT at 9–12 weeks and 6 months of age. Two-way ANOVA showed significant main effects of age (*p* < 0.0001) and genotype (*p* < 0.0001) and there was an interaction between age and genotype (*p* < 0.0001). A Sidak’s *post hoc* analysis indicated a significant difference between WT and ITSN1-LKO mice at both ages and the WTs at 6 months of age showed enhanced LTP compared with their 9–12 weeks old counterparts. There was no difference between 9–12 weeks and 6-months-old LKO mice. Error bars show means ± SEM, *n* = 5 per genotype. ns, not significant.

LTP is a use-dependent neurophysiological process enhancing the strength of the synaptic connection, with hippocampal CA1 region LTP directly correlating with episodic memory acquisition (Nabavi et al., 2014). Due to the fact that the defects in LTP are associated with impaired learning and memory (McNaughton et al., 1986; Chapman et al., 1999) and the performance in the MWM was adversely affected by the deletion of the ITSN1 long isoform, LTP was measured in our study. An impaired LTP was evident in both young and older ITSN1-LKO mice (Figure 4A, *p* < 0.05; Figure 4B, *p* < 0.0001). When we compared the average change in LTP across the four data sets, there were significant main effects of age (*p* < 0.0001) for the WT and genotype (*p* < 0.0001) at both ages. The WTs showed enhanced LTP at 6 months of age compared with their 9–12 weeks old counterparts, while 9–12 weeks and 6-months old LKO mice were equally incapable of sustaining potentiation. ITSN1-LKO mice displayed diminished LTP compared with the WTs at both ages (Figure 4C).

### Deletion of the ITSN1 Long Isoform Had No Effect on Basal MAPK/ERK Activation but Reduced the Activation of Basal AKT

**Figure 5:**
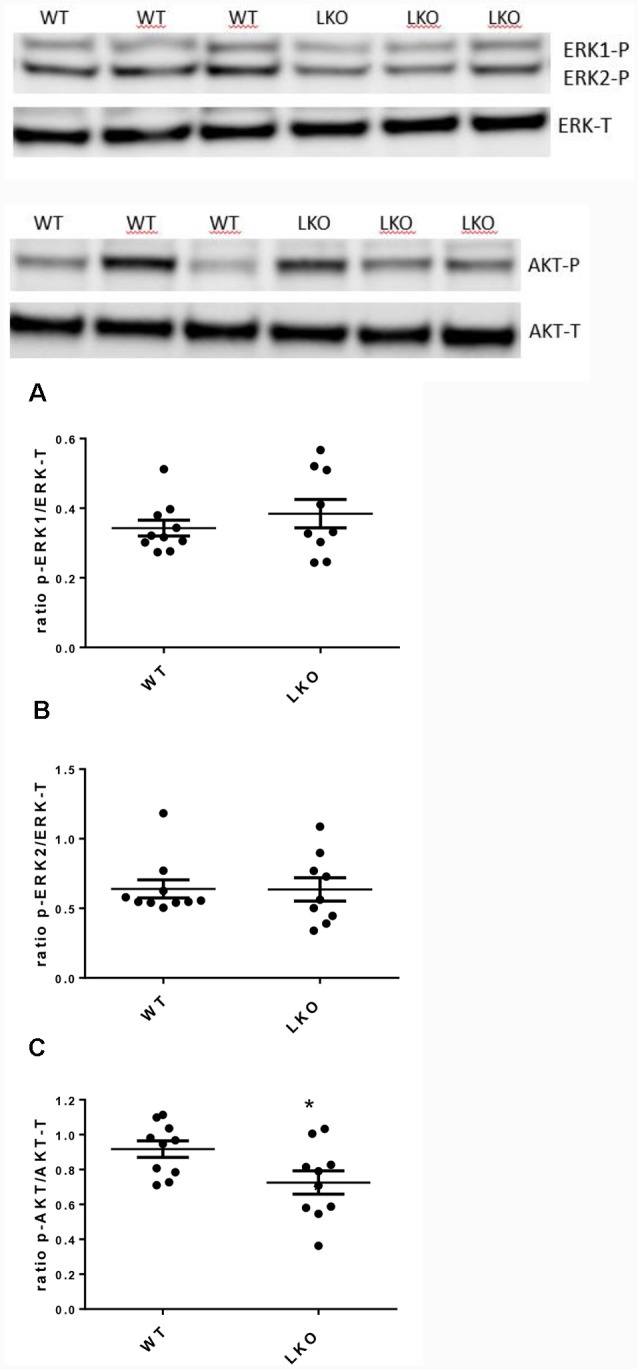
Active mitogen-activated protein kinase (MAPK) was unchanged in the ITSN1-LKO hippocampus but AKT activation was reduced. MAPK and AKT activities were measured by western blotting in mouse hippocampal homogenates. Levels of phosphorylated ERK1 and 2 were measured to determine basal MAPK cell signaling activity. Phosphorylated AKT was measured to determine basal AKT cell signaling activity. Representative Western blots are shown. In 6-months-old mice, no differences were observed for the activated forms of ERK1/2 compared with the WT **(A,B)**. The only difference was a modest decrease in the activity of AKT in LKO mice compared with the controls (**C**; *p* = 0.0306, *n* = 10 per genotype, unpaired two-tailed *t*-test). The data are presented as mean ± SEM. *p < 0.05.

Activation of ERK/MAP kinase signaling is required for the induction of LTP and the CREB-mediated transcriptional events associated with neuronal plasticity, learning and memory consolidation (Impey et al., 1998, 1999). Similarly, AKT1 phosphorylation is required for the expression of LTP and for regulating activity-induced protein synthesis that supports LTP (Bruel-Jungerman et al., 2009; Levenga et al., 2017). Since both LTP and hippocampal-dependent behavior were adversely affected by the loss of the ITSN1 long isoform we investigated the possibility that defects in MAPK and AKT signaling were responsible. We measured the levels of activated (p-ERK1/2) proteins as an indicator of MAPK cell signaling activity and p-AKT as a marker for AKT cell signaling activity. In 6-months-old LKO mice, the levels of active ERK1/2 were no different from WT (Figures 5A,B) but the amount of activated AKT was reduced in ITSN1-LKO hippocampi (Figure 5C, *p* = 0.0306), although the reduction represented a modest 19% decrease over WT.

### Decreased Dendritic Spine Density and Morphological Anomalies in ITSN1-LKO Mice

**Figure 6:**
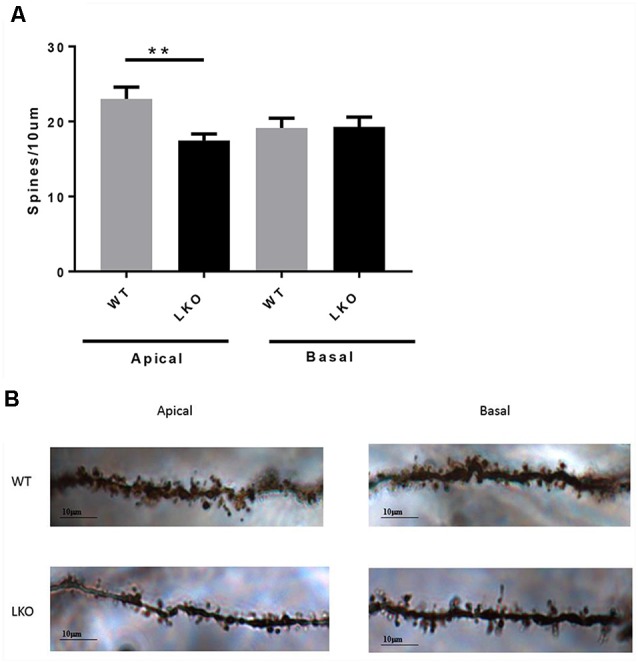
ITSN1-LKO hippocampal pyramidal neurons exhibited decreased dendritic spine density. As shown in **(A)** spine density was significantly decreased on apical dendrites of ITSN1-LKO pyramidal neurons compared with controls (*n* = 3, *p* = 0.0024, unpaired two-tail *t*-test). Spines on basal dendrites were unaffected. **(B)** Representative image of ITSN1-LKO and WT Golgi-stained apical dendrites showing spines. Images were captured with a 100× oil immersion lens. **p < 0.01.

Finally, to investigate whether a lack of the intersectin-1 long isoform has an effect on spine morphogenesis we analyzed dendritic spine density. We counted spines on both apical and basal dendrites on CA1 pyramidal neurons. Compared with WT controls, ITSN1-LKO mice displayed a 24% decrease in apical spine density (*p* < 0.05) but there was no change in the density of spines on basal dendrites (Figure 6).

## Discussion

The aim of this study was to extend our previous work on the biological function of intersectin-1 using knockout mice that we had generated lacking the intersectin-1 long isoform. We report that the deletion of ITSN1-L results in functional deficits in the brain. Specifically, we observed defects in hippocampal-dependent learning and long-term memory which correlated with a reduced capacity to maintain LTP and abnormalities in dendritic spine formation.

To understand the function of ITSN1 long isoform in relation to the spatial and long-term memory, we employed the MWM test. The results showed that ITSN1-LKO mice had spatial learning deficits at 9–12 weeks and 6 months of age. For both age groups, the time taken for ITSN1-LKO mice to find the platform was unchanged after 6 days of training indicating that these mice did not learn the location of the platform.

The probe trial test, however, indicated that the younger ITSN1-LKO mice were able to remember which quadrant the platform had been located in during training but older mice had lost this ability, suggesting an age-related decline in memory formation. This finding is intriguing given that ITSN1 was found to facilitate Reelin signaling (Jakob et al., 2017), a signaling pathway that regulates neuronal migration and synaptic plasticity in the hippocampus. Reelin has also been shown to associate with Alzheimer’s disease (Botella-López et al., 2006), an age-related neurodegenerative disorder inextricably linked to Down syndrome. We may speculate that perturbations in the expression levels of ITSN1 might contribute to the development of Alzheimer’s disease-like neuropathology and cognitive deficits in Down syndrome. In this context, it would be interesting to conduct a battery of memory tests in aged ITSN1 LKO mice. Our data are also consistent with a study that reported spatial memory deficits in the MWM using mice that lacked both long and short isoforms of ITSN1 (Sengar et al., 2013). Our data suggest the long isoform of intersectin-1 is the predominant form involved in memory and cognition.

It is of note that the 9–12 weeks old ITSN1-LKO mice swam faster than their WT counterparts which may reflect a more anxious state that could affect their ability to learn/remember the task. However, there were no differences between the groups in the open field test suggesting that ITSN1-LKO mice are no more anxious than the WT controls. Nevertheless, other anxiety-testing tasks could be performed in further addressing this issue.

It has been established that LTP is an indicator of synaptic plasticity and the fundamental basis of memory consolidation (reviewed in Lynch, 2004). Consistent with the cognitive deficits observed, LTP was decreased in ITSN1-LKO mice compared with the WT for both 9–12 weeks and 6 months age groups. Notably, two recent studies using complete loss-of-function mutants of ITSN1 found no changes in hippocampal LTP compared with control mice (Sengar et al., 2013; Jakob et al., 2017). This is at odds with our data—if the loss of the long ITSN1 isoform causes LTP defects, a similar disruption may be expected with the loss of both isoforms. Whilst the reasons for these discrepancies are unclear, there are a number of possible explanations. First, the expression patterns of ITSN1 and ITSN2 overlap, so perhaps in some instances, ITSN2 can compensate for a complete loss of ITSN1. However, given that a double ITSN1/ITSN2 knockout has normal LTP (Sengar et al., 2013) this explanation seems unlikely. Second, the differences may be a reflection of the distinct experimental models used or differences in experimental parameters, methods of tissue preparation or detection methods, all of which may contribute to differences in outcome measures, or perhaps it is the method of stimulation to induce LTP or the number of pulses which determines the magnitudes of late potentiation (Hernandez et al., 2005). The literature shows that theta-burst stimulation (TBS; Larson and Munkácsy, 2015) is a more efficient method of inducing LTP in comparison with other forms of stimulation for LTP induction and activation of downstream signaling cascades. While we used the more traditional form of stimulation; the high-frequency tetanic stimulation (three trains of 50 pulses at 100 Hz lasting for 500 ms at 20-s intervals) in our studies, this raises the question of what are the processes differentially activated by the different stimulation protocols. Unfortunately, we cannot answer this intriguing question in our mice.

Another explanation could be the age of the mice as some abnormalities would be manifested at a later age. Maybe we would have observed abnormalities in MAPK/ERK activation pathway if we had 12 months old ITSN1-LKO mice.

For example, it has been established that DS is the most common intellectual disability and the most AD-related syndrome. However, it is interesting that over the years, most of the drugs that are used to improve cognition are not related to chromosome 21 genes and tested in 4–6-months-old mice, so the effect of those drugs on the older mice are not even known.

As just mentioned, most DS mouse models have been studied at 4–6 months of age similar to this study. In comparison, where the 12 months old widely used Ts65Dn mouse model was studied by Ahmed et al. (2017), it was revealed that there were differences between younger (6 months) and older (12 months) mice. For example, the abnormalities in the cortex were increased with age, while the abnormalities in the cerebellum decreased. The effect of age on protein abnormalities in the brain of the mouse model of Ts65Dn was also tested in Ahmed et al. (2017). Of relevance, one of the measured proteins was ITSN1 which was increased with age. Furthermore, they showed that ITSN1 was 80% higher in older Ts65Dn mouse compared with the age-matched controls. This raises the question that if we had 12 months old ITSN1-Tg mice, could we have observed changes in the brain and their behavior as the result which we did not see in the 6 months old? This remains to be explored.

Another explanation could be that although the MAPK/ERK pathway signaling could be activated *via* different factors, all lead to transduction of a signal to small GTP-binding proteins (Ras, Rap1) which in turn would activate the MAPK/ERK pathways (Goldsmith and Dhanasekaran, 2007). For example, a variety of ligands could activate G protein-coupled receptors (GPCRs). Consequently, the receptor activation induces the G protein to exchange GDP for GTP, causing the dissociation of the GTP-bound α and β/γ subunits and initiating the signaling cascades (Tuteja, 2009). Receptors coupled to different heterotrimeric G protein subtypes can employ different scaffolds to trigger the small G protein/ MAPK cascade, utilizing different classes of Tyr kinases (Belcheva and Coscia, 2002). Src family kinases are engaged following the stimulation of PI3Kγ by β/γ subunits. They are also recruited by receptor internalization, and activation of receptor Tyr kinases, or by signaling through an integrin scaffold involving proline-rich tyrosine kinase 2 (Pyk2) and/or focal adhesion kinase (FAK; Sun et al., 2008). GPCRs can also employ phospholipase Cβ (PLCβ) to initiate activation of PKC and Calcium/calmodulin protein kinase II (CaMKII), which can either inhibit or activate the downstream MAPK pathway (Goldsmith and Dhanasekaran, 2007). We did not look at any of these pathways that lead to MAPK/ERK activation. It is possible that if we examined one of these pathways, we could observe the changes that would be the cause of LTP deficits.

Despite our finding that a lack of the intersectin-1 long isoform was associated with deficits in LTP and in learning and memory, this could not be explained by a change in basal MAPK/ERK signaling. Numerous studies have been conducted in EGF-stimulated cell lines where ITSN1 has either been exogenously added (Adams et al., 2000; Xie et al., 2008) or silenced using shRNAs (Martin et al., 2006; Predescu et al., 2007; Russo et al., 2015). These studies variously report reduced ERK1/2 activation, increased ERK1/2 activation or no change, depending on the model used. In the context of these results, it would be informative to examine the activation status of MAPK signaling pathways after induction of LTP in isolated ITSN1-LKO hippocampal slices or in cultured hippocampal neurons using chemically induced LTP. To the best of our knowledge, there is only a single report examining MAPK/ERK signaling in mice where the expression of ITSN1 has been manipulated. In this study, a lung ITSN1 knockdown was generated by the repeated delivery of a specific ITSN1-targeting siRNA (Bardita et al., 2013). Acute depletion of ITSN1 diminished MAPK/ERK signaling but chronic depletion of ITSN1 resulted in reactivation of MAPK/ERK signaling due to increased cell proliferation and repair of the injured lungs.

Although we demonstrated no change in MAPK/ERK signaling activity in ITSN1-LKO mice, we did find a decrease of 19% in the basal level of phosphorylated AKT in ITSN1-LKO hippocampal homogenates. The involvement of AKT signaling in LTP is well established (Bruel-Jungerman et al., 2009; Levenga et al., 2017). Indeed, the use of AKT inhibitors or ablation of *Akt1* in mice impaired L-LTP, the long-lasting form of LTP whose maintenance is required for forming memories (Levenga et al., 2017). We demonstrate an LTP impairment in our ITSN1-LKO mice, suggesting that, at least in part, this may be a result of the negative effect that the lack of ITSN1-L has on the activation of AKT. In support of this notion is a study that showed depletion of ITSN1 in EGF-stimulated neuroblastoma cells resulted in a 50% reduction in the activation of AKT (Russo et al., 2015). Furthermore, in our previous study, we have shown that ITSN1-LKO mice have vesicle-trafficking abnormalities (Yu et al., 2008) which could explain the deficits in their LTP. It has been established that recycling endosomes transport AMPA receptors to the plasma membranes for LTP (Park et al., 2004).

Examination of dendritic spine numbers and density in hippocampal pyramidal neurons revealed a 24% reduction in apical spine density in ITSN1-LKO mice compared with controls. Our results, using an *in vivo* model, agree with other ITSN1 over-expression, knockdown and dominant-negative studies showing dendritic spine abnormalities (Hussain et al., 2001; Irie and Yamaguchi, 2002; Thomas et al., 2009). Examination of hippocampal dendritic spines in our total ITSN1 knock out mice revealed that the density of both basal and apical dendritic spines have been decreases (data not shown; Yu, 2010). Interestingly, however, Jakob et al. (2017) did not observe abnormalities in hippocampal dendritic spine formation in their total ITSN1 knockout. Thus, it appears, for reasons currently unknown, that the complete loss of ITSN1 preserves spine integrity, yet the loss of the neuron-specific long isoform results in a disruption to dendritic spine formation.

Although the molecular mechanisms governing spine morphogenesis are not fully understood, factors including actin filament rearrangement (Matus, 1999, 2000) and the level of synaptic activity and plasticity (Engert and Bonhoeffer, 1999; Maletic-Savatic et al., 1999) have been implicated. EphB receptors and Cdc42-mediated actin polymerization are responsible for dendritic spine development and they are functionally dependent on the recruitment of ITSN1-L to EphB2/syndecan-2 co-clusters (Irie and Yamaguchi, 2002). Based on our study, we suggest that the absence of ITSN1-L has led to a decrease of spine number, possibly due to a deficit in complex formation between ITSN1 and EphB2-Cdc42. We cannot however, exclude the possibility that other functions influenced by the absence of ITSN1-L might also have an adverse effect on spine development and number. For example, synapse dysfunction and synaptic structural changes are accompanied by decreased spine number (Zhang et al., 2005) and we have shown that synaptic vesicle recycling is defective in ITSN1 null mice (Yu et al., 2008).

Finally, the finding that a difference exists between apical and basal spine number on dendrites of the same neuron is not unusual. Indeed in adult GABAA receptor α1 subunit KO mice, only apical spine numbers on pyramidal cells located in the visual cortex were decreased (Heinen et al., 2003). Neither, Heinen et al. (2003), nor we, are able to explain the molecular bases for this phenomenon and the functional/biological implications of having an imbalance in apical vs. basal spine density also remain unclear.

In summary, we have shown that a lack of ITSN1-L disrupts a number of processes, including LTP, dendritic spine development and hippocampal-dependent learning and memory. Our earlier study demonstrated that mice deficient for the long isoform of ITSN1 exhibit disturbances in synaptic vesicle trafficking and endocytic function in the brain (Yu et al., 2008). Taken together, our data suggest that ITSN1-L is required for normal brain function and specifically, for learning and spatial (long-term) memory. How dysregulation of ITSN1 expression may contribute to the neural phenotypes characteristic of Down syndrome remains elusive but studies such as ours take us a step further along the pathway of unraveling the molecular mechanisms responsible.

## Data Availability Statement

The datasets generated for this study are available on the request to the corresponding authors.

## Ethics Statement

The animal study was reviewed and approved by the Florey Institute of Neuroscience and Mental Health Animal Ethics Committee and were conducted in accordance with the Australian Code of Practice for the Care and Use of Animals for Scientific Purposes as described by the National Health and Medical Research Council of Australia.

## Author Contributions

NM designed, carried out experiments, collected the data, analyzed and wrote the manuscript. PA, DF and MP designed, supervised and edited the manuscript. FC carried out and analyzed electrophysiology experiments. YY designed, carried out and analyzed dendritic spines experiments. CS assisted with behavioral experiments and data acquisition.

## Conflict of Interest

The authors declare that the research was conducted in the absence of any commercial or financial relationships that could be construed as a potential conflict of interest.
